# Obesity impairs lactation performance in mice by inducing prolactin resistance

**DOI:** 10.1038/srep22421

**Published:** 2016-03-01

**Authors:** Daniella C. Buonfiglio, Angela M. Ramos-Lobo, Vanessa M. Freitas, Thais T. Zampieri, Vanessa S. Nagaishi, Magna Magalhães, Jose Cipolla-Neto, Nathalie Cella, Jose Donato Jr.

**Affiliations:** 1 Department of Physiology and Biophysics, Institute of Biomedical Sciences, University of São Paulo, São Paulo, SP, 05508-000, Brazil; 2 Department of Cell and Developmental Biology, Institute of Biomedical Sciences, University of São Paulo, São Paulo, 05508-000, Brazil

## Abstract

Obesity reduces breastfeeding success and lactation performance in women. However, the mechanisms involved are not entirely understood. In the present study, female C57BL/6 mice were chronically exposed to a high-fat diet to induce obesity and subsequently exhibited impaired offspring viability (only 15% survival rate), milk production (33% reduction), mammopoiesis (one-third of the glandular area compared to control animals) and postpartum maternal behaviors (higher latency to retrieving and grouping the pups). Reproductive experience attenuated these defects. Diet-induced obese mice exhibited high basal pSTAT5 levels in the mammary tissue and hypothalamus, and an acute prolactin stimulus was unable to further increase pSTAT5 levels above basal levels. In contrast, genetically obese leptin-deficient females showed normal prolactin responsiveness. Additionally, we identified the expression of leptin receptors specifically in basal/myoepithelial cells of the mouse mammary gland. Finally, high-fat diet females exhibited altered mRNA levels of ERBB4 and NRG1, suggesting that obesity may involve disturbances to mammary gland paracrine circuits that are critical in the control of luminal progenitor function and lactation. In summary, our findings indicate that high leptin levels are a possible cause of the peripheral and central prolactin resistance observed in obese mice which leads to impaired lactation performance.

Prolactin is a protein secreted by the anterior pituitary gland in response to various stimuli, such as stress, mating, nursing and ovulation^1^. Although prolactin may have numerous biological effects^1^, this hormone is best known for its stimulatory actions on mammary gland development and milk production^2^,^3^. Consequently, prolactin is typically associated with the lactation period. In addition to the mammary gland, the brain also expresses significant amounts of the prolactin receptor (PrlR). Prolactin-responsive neurons are abundant in the hypothalamus^4^,^5^,^6^, especially in the preoptic region, which is an important site for the regulation of maternal behaviors^7^,^8^. In fact, classical studies have found stimulatory effects of prolactin on maternal behavior expression^9^,^10^. Accordingly, PrlR knockout mice exhibit robust defects in maternal behavior^11^.

Several lines of evidence also indicate that prolactin is a hormone capable of modulating energy balance. Hyperprolactinemia caused either by dopamine-inhibiting drugs or pituitary tumors predisposes animals to obesity^12^,^13^,^14^. Selective disruption of dopamine D2 receptors in pituitary lactotrophs leads to hyperprolactinemia, increased body weight, and adiposity in female mice^15^. Conversely, PrlR knockout mice have a lean phenotype^16^. Some authors have also highlighted the contribution of prolactin to pregnancy-induced hyperphagia^17^. This effect may involve cross-talk between prolactin and leptin, which ultimately leads to central leptin resistance. For example, prolactin administration increases the hypothalamic expression of suppressor of cytokine signaling (SOCS) proteins^18^. Because SOCS proteins inhibit leptin signaling^19^, and subsets of leptin receptor (LepR)-expressing neurons are responsive to prolactin^6^, high levels of prolactin during pregnancy and lactation may decrease leptin sensitivity. Accordingly, chronic intracerebroventricular infusion of prolactin blocks the anorexigenic effects induced by an acute leptin injection^20^,^21^. In addition, SOCS3 inactivation in LepR-expressing cells increases leptin sensitivity and reduces food intake and weight gain during pregnancy and lactation^22^.

Based on the aforementioned information, pathological or physiological (pregnancy and lactation) hyperprolactinemia may interfere with leptin signaling, which indicates the potential for cross-talk between prolactin and leptin. Interestingly, obesity, which is a hyperleptinemic condition, has negative effects on some biological functions regulated by prolactin. Several studies have shown a lower breastfeeding success and decreased lactation performance associated with obesity^23^,^24^,^25^,^26^,^27^,^28^,^29^,^30^,^31^. Obesity delays the initiation of lactation, reduces lactation duration and favors the premature introduction of non-breast milk foods and fluids^23^,^24^,^25^,^26^,^27^,^28^,^29^,^30^,^31^. Several hypotheses have been suggested to explain the lower lactation success in obese women, including weaker prolactin responses to suckling, smaller decreases in progesterone levels in the postpartum period, decreased circulating human placental lactogen levels, alterations in suckling patterns in infants born to mothers with gestational metabolic imbalances, psychological issues, increased serotonin production within the mammary gland leading to an inflammatory process, and mechanical problems due to excessive adipose tissue deposition in the mammary tissue^28^,^29^,^30^,^31^,^32^. However, further studies are required to test these possibilities.

In the present study, we investigated an alternative hypothesis to explain the reduced lactation success in obese individuals. In this regard, increased cytokine levels (e.g., leptin) in obese animals may cause prolactin resistance. Therefore, our objective was to evaluate the lactation performance and maternal behaviors of obese mice, as well as the sensitivity to prolactin in the mammary tissue and hypothalamus and the responsiveness to leptin in the mammary gland. Given the high incidence of obesity in the global population^33^ and the important beneficial effects of breastfeeding for the health of infants and nursing mothers^24^,^29^,^30^,^34^,^35^, a better comprehension of the factors that may impair lactation is of paramount importance for public health.

## Results

### Characterization of high-fat diet (HFD)-induced obesity

**Figure 1:**
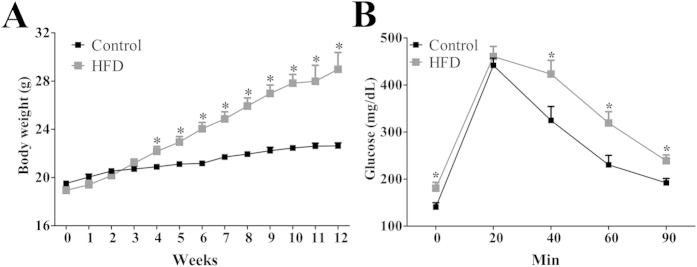
Characterization of HFD-induced obesity. (**A**) Weekly body weight of control (*n* = 34) and HFD (*n* = 44) groups. (**B**) Glucose tolerance test (2 g glucose/kg; i.p.) in control (*n* = 8) and HFD (*n* = 12) mice after 12 weeks on each diet. *p < 0.05 compared to control group.

To evaluate the efficacy of a HFD to induce obesity, the body weight of control and HFD females was monitored weekly. HFD consumption led to a significant increase in body weight after 4 weeks (*p* < 0.05), and this difference was maintained (Fig. 1A). To determine whether chronic HFD intake affects glucose homeostasis in female mice, control and HFD animals were subjected to a glucose tolerance test (GTT) after 12 weeks on either diet. HFD females exhibited higher glucose levels at baseline and an impaired glucose tolerance during the GTT compared to control animals (*p* < 0.05; Fig. 1B). Overall, these results confirmed that chronic HFD intake leads to obesity and glucose intolerance in female C57BL/6 mice.

### Diet-induced obesity impairs offspring viability, milk production and mammopoiesis

**Figure 2:**
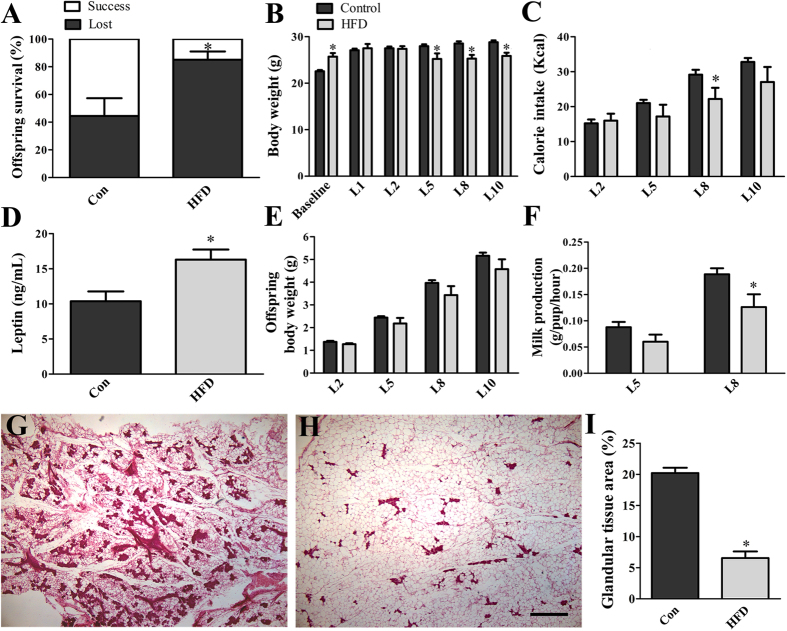
Diet-induced obesity impairs offspring viability, milk production and mammopoiesis. (**A**) Offspring survival in primiparous control (*n* = 26) and HFD (*n* = 44) females. (**B**) Body weight changes in the dams before pregnancy (baseline) and during lactation (*n* = 7–14/group). (**C**) Daily calorie intake of the dams during lactation (*n* = 7–14/group). (**D**) Serum leptin levels at day 10 of lactation (*n* = 7–14/group). (**E**) Offspring body weight (*n* = 7–14/group). (**F**) Milk production at days 5 and 8 of lactation (*n* = 7–14/group). (**G,H**) Representative photomicrographs illustrating hematoxylin-eosin staining of mammary gland of control (**G**) and HFD (**H**) females in late pregnancy. (**I**) Quantification of the glandular tissue area of control and HFD females. *p < 0.05 compared to the control group. Scale bar = 500 μm.

After inducing obesity, the females were bred. Those that did not become pregnant or that had miscarriages were excluded from the experiment. Initially, we investigated the females’ capacity to sustain the offspring after birth (Fig. 2A). Notably, 85 ± 6% of HFD females that gave birth lost their offspring after a few days in contrast to 44 ± 13% of control females (*p* = 0.0447). The apparent reason for the offspring loss in both groups was a lack of maternal care and lactation. For those offspring that survived, we found no difference in the number of pups per litter between control (6.9 ± 0.3) and HFD (6.0 ± 0.7; *p* = 0.1887) females. Before mating (baseline), HFD females were significantly *(p* < 0.0001) heavier than control animals (Fig. 2B). However, the body weight difference between control and HFD groups was no longer present during the first days of lactation, and surprisingly, the HFD females exhibited a reduction in body weight from day 5 to day 10 of lactation (*p* < 0.05; Fig. 2B). The body weight shift may be explained by a lower calorie intake of HFD females during lactation than that of controls (*p* < 0.005; Fig. 2C). Nevertheless, HFD females still exhibited higher circulating leptin levels (*p* < 0.05) at day 10 of lactation than control females (Fig. 2D).

To investigate the effects of maternal obesity on the offspring, we analyzed the growth pattern of pups. We found no difference in the body weight of pups born to control and HFD dams during the analyzed period, although we observed a slight trend (*p* = 0.0916) towards a reduction in HFD-born pups throughout the analyzed period (Fig. 2E). Milk production was not different at day 5 of lactation (L5; *p* = 0.1354), but when the demand for milk increased at L8, HFD females showed a lower milk production than control mice (*p* = 0.0159; Fig. 2F). Histological analyses on late-pregnant animals revealed markedly less mammary glandular tissue area in HFD females than in controls (*p* < 0.0001; Fig. 2G–I).

### Impaired maternal behaviors in obese mice

**Figure 3:**
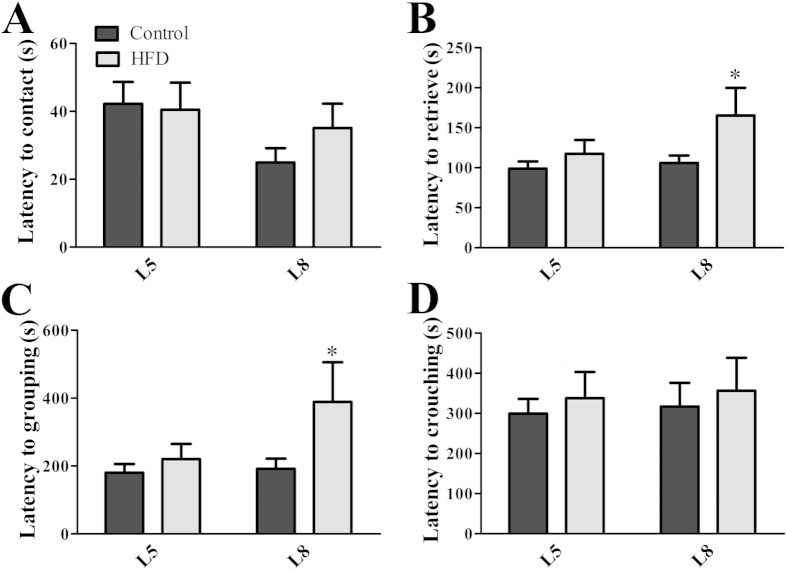
Impaired maternal behavior in primiparous obese mice. (**A–D**) Time required to contact and retrieve all pups, to group them into the nest and to crouch over pups (*n* = 7–14/group) at days 5 and 8 of lactation (L5 and L8, respectively). *p < 0.05 compared to control group.

The infant survival of altricial mammals depends on both the mother’s milk production and the parental care provided during youth^7^,^8^. To investigate whether the lower survival rate of HFD offspring was influenced by changes in maternal behavior, we separated the pups and after their reintroduction into the mother’s cage we determined the time required for the mother to contact them. No significant changes between groups were observed in the latency to contact the pups at L5 (*p* = 0.8720) or L8 (*p* = 0.1973; Fig. 3A). Then, we assessed the latency to retrieve all pups into the nest, to group them and crouch over to initiate feeding. At L5, there was no difference between groups in the expression of these behaviors (Fig. 3B–D); however, at L8 the time required to retrieve and group the pups into the nest was significantly longer in the HFD group than that observed in the control group (*p* < 0.05; Fig. 3B,C). No statistically significant difference was observed in the latency to crouch over the pups (Fig. 3D).

### Reproductive experience minimizes the effects of obesity on lactation and maternal behavior

**Figure 4:**
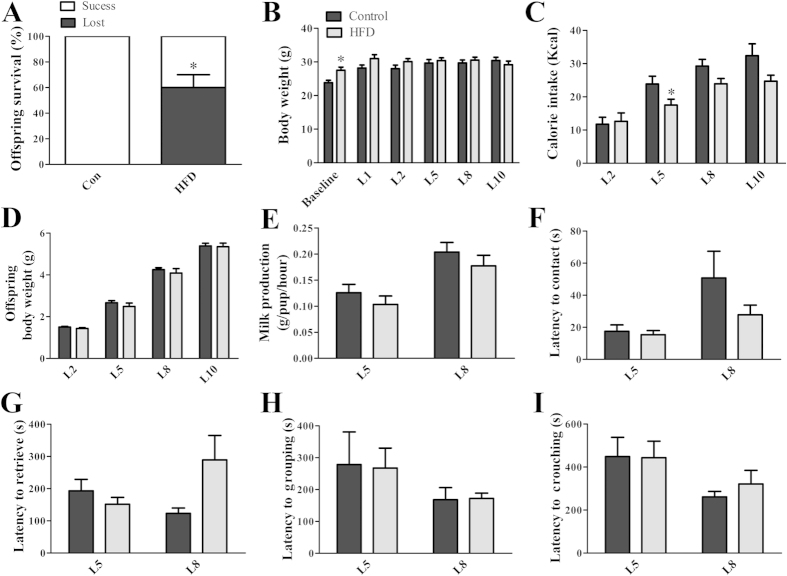
Reproductive experience minimizes the effects of obesity on lactation and maternal behavior. (**A**) Offspring survival rate in the second litter of control (*n* = 7) and HFD (*n* = 21) females. (**B**) Body weight changes in the dams before pregnancy (baseline) and during lactation (*n* = 7–9/group). (**C**) Daily energy intake of the dams during lactation (*n* = 7–9/group). (**D**) Offspring body weight (*n* = 7–9/group). (**E**) Milk production at days 5 and 8 of lactation (*n* = 7–9/group). (**F–I**) Latency to contact and retrieve all pups, to group them into the nest and to crouch over pups (*n* = 7–9/group). *p < 0.05 compared to control group.

Reproductive experience enhances maternal care^36^,^37^ and reduces circulating prolactin levels^38^. In addition, reproductively experienced female rats exhibit increased prolactin responsiveness by showing a greater number of prolactin-induced phosphorylated signal transducer and activator of transcription 5 (pSTAT5) immunoreactive cells in the hypothalamus than virgin animals^39^. However, it is unknown whether reproductive experience improves offspring survival and maternal care in obese mice. Therefore, primiparous control and HFD females that lost their litters were bred again to evaluate lactation performance with their second offspring. Interestingly, only 40 ± 10% of the offspring of obese females survived the first postnatal days compared to a 100% survival rate in the control group (*p* = 0.0039; Fig. 4A). Once again, there was no difference in the number of pups per litter between groups (Control: 5.6 ± 0.5; HFD: 6.2 ± 0.6; *p* = 0.4535). Before the second breeding experience, HFD females were significantly heavier than the controls (*p* = 0.0096; Fig. 4B); however, just after birth, the difference in body weight previously observed between the groups was no longer present (Fig. 4B). Similar to what was observed during the first lactation period, reproductively experienced obese females showed a lower calorie intake during lactation than control animals (*p* < 0.05; Fig. 4C). Nonetheless, these changes did not affect offspring weight gain (Fig. 4D) or milk production (Fig. 4E). Additionally, no changes in maternal behavior expression were observed between HFD and control females in their second litter (Fig. 4F–I).

### Obese females display prolactin resistance in the mammary gland and hypothalamus

**Figure 5:**
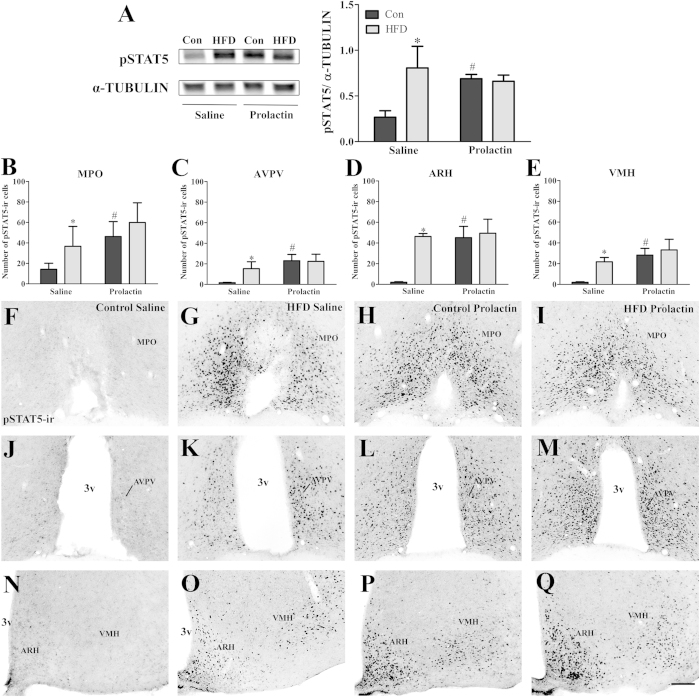
Prolactin responsiveness in the mammary gland and hypothalamus of diet-induced obese females. (**A**) Phosphorylation of STAT5 (pSTAT5) in the mammary gland of control and HFD mice after a saline (*n* = 4/group) or prolactin (*n* = 8/group) injection. (**B–E**) Number of pSTAT5-immunoreactive cells in the MPO (**B**), AVPV (**C**), ARH (**D**) and VMH (**E**) of saline- or prolactin-induced in control and HFD mice. (**F–Q**) Photomicrographs of coronal sections illustrating saline-induced pSTAT5-immunoreactivity (pSTAT5-ir) in control (**F,J,N**) and HFD (**G,K,O**) mice or prolactin-induced pSTAT5-ir in control (**H,L,P**) and HFD (**I,M,Q**) mice in the MPO (**F–I**), AVPV (**J–M**), ARH (**N–Q**) and VMH (**N–Q**). Scale bar = 100 μm. Abbreviations: 3v, third ventricle; fx, fornix. *p < 0.05 HFD saline compared to control saline group. ^#^*p* < 0.005 control prolactin compared to control saline group.

Our findings revealed that biological functions regulated by prolactin signaling, such as milk production and maternal behaviors, are impaired in obese females. Therefore, in an attempt to uncover the mechanisms involved in obesity-induced impaired lactation, we investigated whether obese females present normal responsiveness to prolactin. For this purpose, control and HFD females received a single injection of saline or prolactin, and we assessed the induction of STAT5 phosphorylation in the mammary tissue and central nervous system. Remarkably, saline-injected HFD females exhibited a higher STAT5 phosphorylation in the mammary tissue than saline-injected controls (*p* < 0.05; Fig. 5A). Furthermore, prolactin stimulus in HFD females was unable to increase STAT5 phosphorylation beyond the levels observed in HFD saline-injected animals. In contrast, the prolactin stimulus significantly increased STAT5 phosphorylation in control females compared to saline-injected control mice (*p* = 0.0264; Fig. 5A). Next, we assessed the ability of a peripheral prolactin injection to induce STAT5 phosphorylation in specific hypothalamic nuclei that express the PrlR^4^,^5^,^6^ (Fig. 5B–E). Saline-injected control females exhibited low numbers of pSTAT5 immunoreactive cells in the analyzed areas, including the medial preoptic area (MPO; Fig. 5F), anteroventral periventricular nucleus (AVPV; Fig. 5J), arcuate nucleus of the hypothalamus (ARH; Fig. 5N) and ventromedial nucleus of the hypothalamus (VMH; Fig. 5N). Similar to what was observed in the mammary gland, saline-injected HFD females showed higher numbers of pSTAT5 immunoreactive cells in the MPO (Fig. 5G), AVPV (Fig. 5K), ARH and VMH (Fig. 5O), and importantly, the prolactin stimulus was unable to further increase prolactin responsiveness in these areas (Fig. 5I,M,Q). In contrast, as expected, the prolactin stimulus induced STAT5 phosphorylation in the MPO (Fig. 5H), AVPV (Fig. 5L), ARH and VMH (Fig. 5P) of control females to levels significantly higher than those observed in saline-treated controls (*p* < 0.005). Therefore, diet-induced obesity led to prolactin resistance in the mammary tissue and hypothalamus.

### Leptin is required to induce prolactin resistance in obese mice

**Figure 6:**
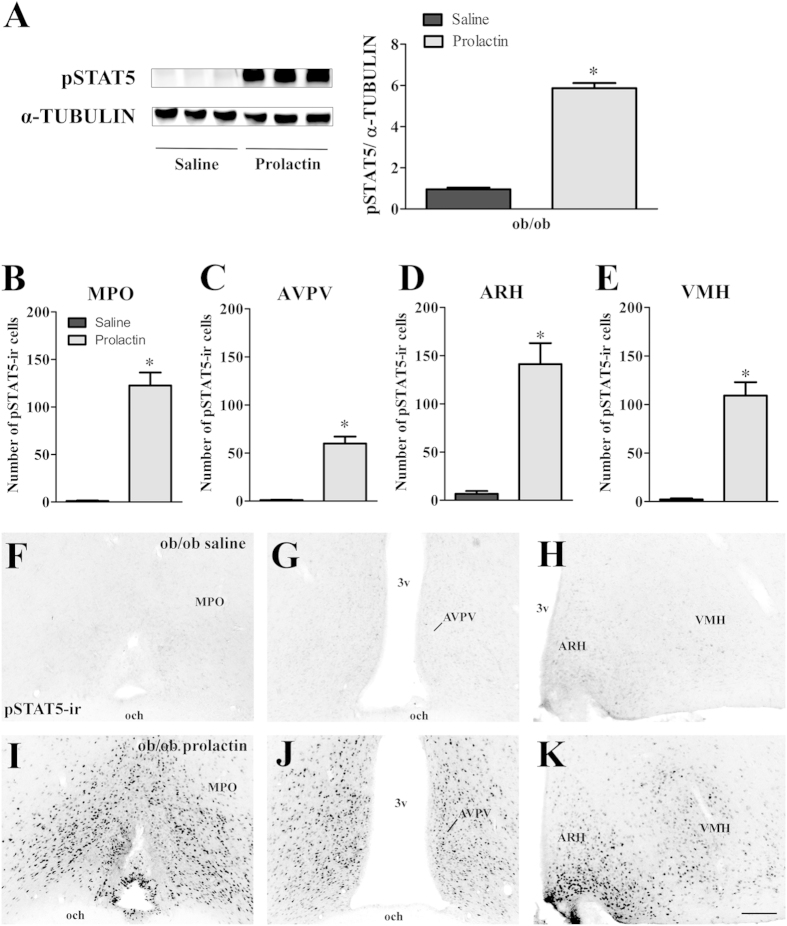
Prolactin sensitivity test in the mammary gland and hypothalamus of ob/ob females. (**A**) pSTAT5 in the mammary gland of saline- or prolactin-treated ob/ob females (*n* = 5/group). (**B–E**) Number of pSTAT5-immunoreactive cells in the MPO (**B**), AVPV (**C**), ARH (**D**) and VMH (**E**) of saline- or prolactin-injected ob/ob mice. (**F–K**) Photomicrographs of coronal sections illustrating saline-induced pSTAT5-ir (**F–H**) or prolactin-induced pSTAT5-ir (**I–K**). Scale bar = 100 μm. *p < 0.0001.

So far, our findings indicate that obesity leads to prolactin resistance during lactation. However, many factors are altered in obese animals and could contribute to this condition. Based on the evidence that leptin acts in major prolactin-responsive tissues^6^,^40^,^41^,^42^, we investigated the possible participation of leptin in the prolactin resistance induced by obesity. For that purpose, we repeated the prolactin sensitivity test performed in HFD-induced obese mice, but now using genetically obese leptin-deficient (ob/ob) female mice (body weight: 48.8 ± 1.0 g; Fig. 6). We compared STAT5 phosphorylation levels in the mammary gland and hypothalamus between saline and prolactin injected ob/ob females. Unlike what we observed in HFD-induced obese mice, ob/ob females exhibited low basal pSTAT5 levels in the mammary gland, and prolactin was able to induce a robust response by increasing pSTAT5 levels six times (*p* < 0.0001; Fig. 6A). Saline-injected ob/ob females also exhibited very low pSTAT5 levels in the hypothalamus (Fig. 6B–K), and importantly, prolactin induced a pronounced increase in the number of pSTAT5-immunoreactive cells (Fig. 6B–K). The differences in prolactin responsiveness between HFD-induced and leptin-deficient obese mice suggest that leptin plays a key role in inducing prolactin resistance in obese mice.

### The mammary gland is directly responsive to leptin

**Figure 7:**
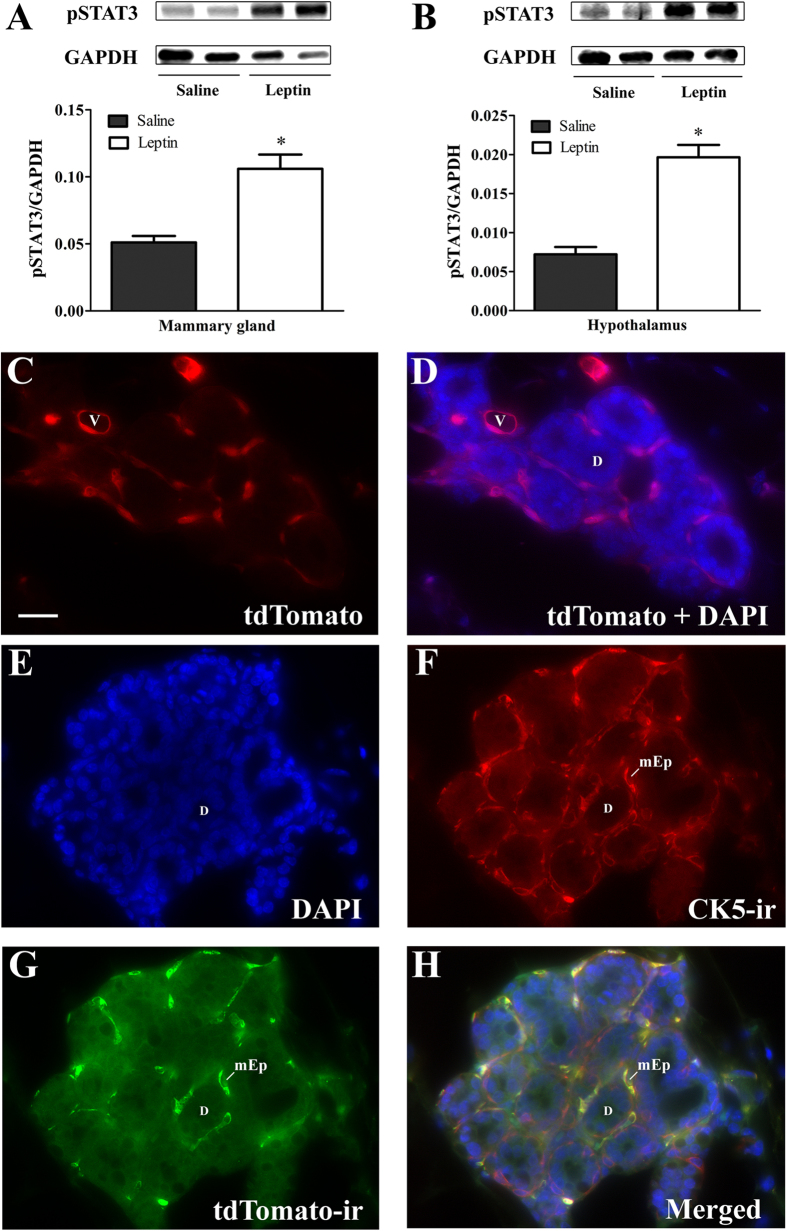
Leptin responsiveness and localization of leptin receptor in the mammary gland of female mice. (**A**) Phosphorylation of STAT3 (pSTAT3) in the mammary gland of mice induced by a saline or leptin injection (*n* = 5/group). (**B**) pSTAT3 in the hypothalamus induced by saline or leptin. (**C**) Photomicrograph illustrating the expression of the tdTomato fluorescent protein. (**D**) Merged picture showing the expression of the tdTomato fluorescent protein and epithelial cell nuclei stained with DAPI fluorescence. (**E–H**) Co-localization of DAPI, CK5 and tdTomato in myoepithelial cells of the mammary gland. Abbreviations: d, lactiferous duct; mEp, myoepithelial cells; v, blood vessel. Scale bar = 50 μm. *p < 0.005 compared to saline group.

Recently, our group demonstrated that LepR is expressed in specific populations of prolactin-responsive cells in the brain^6^. This co-expression suggests a possible interaction between leptin and prolactin to control the activity of these neuronal cells. However, it remains unclear whether the mammary tissue of mice is directly responsive to leptin. To assess the ability of leptin to activate LepR signaling pathways in mammary tissue, female mice received an acute leptin injection, and we assessed the phosphorylation of STAT3 (pSTAT3) as a marker of leptin responsiveness^6^,^22^,^43^,^44^. We found a robust STAT3 phosphorylation induced by leptin in the mammary gland of female mice (*p* < 0.005; Fig. 7A). Notably, the magnitude of this activation was similar to that observed in the hypothalamus, which is a well-known leptin-responsive tissue (*p* < 0.0001; Fig. 7B). These findings indicate that leptin is capable of activating the long form of LepR in the mammary tissue of mice.

### The leptin receptor is expressed in myoepithelial cells of the mammary gland

Previous studies in ovine and bovine found LepR expression in mammary epithelial cells^40^,^41^,^42^. However, information regarding such expression patterns in rodents is scarce. To elucidate the sites of action of leptin in the mouse mammary gland, we analyzed LepR localization using a LepR-reporter mouse that expresses the red fluorescent protein tdTomato only in cells that contain the long form of LepR, as previously described^6^,^45^. Initially, we analyzed the expression of tdTomato without additional staining (Fig. 7C,D). The tdTomato protein was observed around the acini between luminal epithelial cells and the stroma and in endothelial cells (Fig. 7C,D). Because of this peculiar distribution, we performed a co-localization between tdTomato and cytokeratin 5 (CK5), which is a marker for myoepithelial cells (Fig. 7E–H). We found that the vast majority of LepR-expressing cells in the mammary gland are positive for CK5. Therefore, based on the data obtained using our LepR-reporter mouse, LepR is specifically expressed in basal/myoepithelial cells of the mammary gland.

### HFD alters the expression of key transcripts that regulate mammary gland function

**Figure 8:**
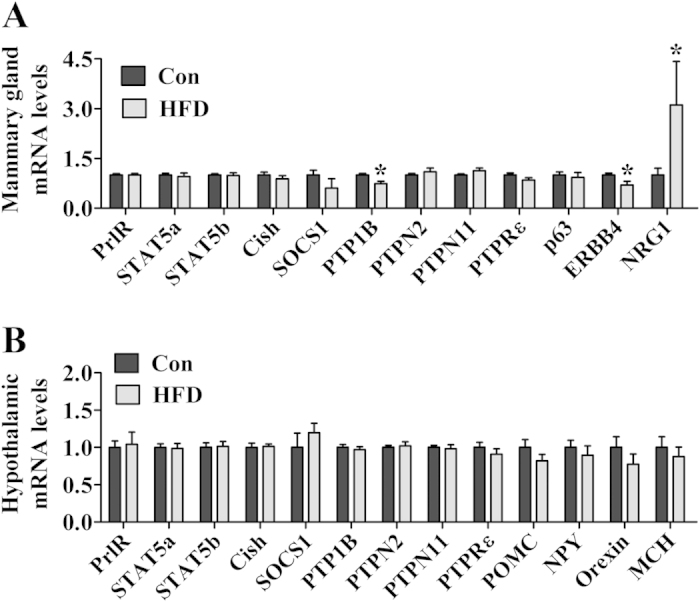
Gene expression analyses in the mammary gland and hypothalamus of control (*n* = 7) and HFD (*n* = 4) lactating females. The females were euthanized at day 10 of lactation, had free access to food and were kept with their litters until the time of euthanasia. Total RNA from the inguinal mammary gland (**A**) and whole hypothalamus (**B**) were used for reverse transcription followed by real-time PCR analyses. The data are reported as fold change compared to values obtained from the control group (set at 1.0). *p < 0.05 compared to control group.

To investigate possible molecular mechanisms involved in the defects presented by HFD females, we performed a gene expression analysis in the mammary tissue and hypothalamus of mice at day 10 of lactation. We analyzed several transcripts in the mammary tissue and found reduced PTP1B mRNA levels in HFD females (Fig. 8A). Additionally, we investigated the expression of import genes recently described as part of a basal-to-luminal paracrine circuit that controls luminal progenitor function and lactation^46^. Although no changes in p63 expression were observed between groups, HFD females exhibited altered levels of ERBB4 and NRG1 (neuregulin-1) mRNA compared to control mice (Fig. 8A). In the hypothalamus, we found no significant changes in the expression of any of the transcripts assessed, including those encoded by genes involved in prolactin signaling, SOCS proteins, protein-tyrosine phosphatases (PTPs) and neuropeptides related to energy balance regulation (Fig. 8B).

## Discussion

Obesity reduces breastfeeding success and lactation performance in women^23^,^24^,^25^,^26^,^27^,^28^,^29^,^30^,^31^. However, the mechanisms involved have not been completely identified. In the present study, we first confirmed that obesity impairs lactation in mice. We found reduced milk production and offspring viability, as well as impaired mammopoiesis and postpartum maternal behaviors, in HFD females. Next, we proposed a novel mechanism to explain the reduced lactation success caused by obesity. Because cytokine signaling causes intrinsic negative feedback mechanisms^19^, high cytokine levels in obese individuals could impair PrlR signaling. Therefore, we hypothesized that obese mice may exhibit prolactin resistance. In accordance with our initial hypothesis, the mammary tissue and hypothalamus of HFD mice were unresponsive to a prolactin stimulus. Importantly, we demonstrated that despite being obese, leptin-deficient females exhibited normal prolactin responsiveness, suggesting that leptin is an essential factor for inducing prolactin resistance in obese animals. Interestingly, basal levels of STAT5 phosphorylation were already elevated in both tissues in HFD females. This phenomenon also occurs with leptin signaling, in which obese animals exhibit high basal levels of STAT3 phosphorylation and fail to further increase STAT3 phosphorylation after a leptin stimulus^43^,^44^. As little information on the distribution of LepR in the murine mammary gland is available, we investigated whether leptin induces STAT3 phosphorylation in this tissue and which cells express the LepR. We found that leptin is capable of activating the long form of LepR in the murine mammary gland, which is specifically expressed in basal/myoepithelial cells. Finally, we demonstrated that diet-induced obesity disrupts the expression of key transcripts that regulate mammary gland functioning. Overall, our findings suggest that high leptin levels are a possible cause of the peripheral and central prolactin resistance presented by obese animals which leads to impaired lactation performance.

Prolactin resistance is a condition that needs further characterization. In the few case reports available in the literature, prolactin resistance has been suggested as a possible cause of alactogenesis in patients with a normal pituitary reserve, prolactin dynamics and mammary architecture^47^. However, to the best of our knowledge, prolactin resistance has not previously been associated with obesity. Using STAT5 phosphorylation as a marker of prolactin responsiveness^4^,^5^,^6^,^39^,^48^, we demonstrated that HFD-induced obese mice were unable to respond to prolactin with responses similar to those observed under basal conditions. This defect, associated with the loss of several functions regulated by prolactin, was interpreted as a prolactin resistance condition. Other evidence of the existence of prolactin resistance in obese females was provided by comparing the results obtained from virgin and reproductively experienced animals. Reproductive experience reduces prolactin circulating levels^38^, favors an early onset of maternal care^36^,^37^ and, importantly, increases the responsiveness to a prolactin stimulus^39^. For example, Sjoeholm *et al.*^39^ found a higher number of prolactin-induced pSTAT5 immunoreactive cells in the hypothalamus of primiparous rats than in virgin animals. Therefore, reproductive experience enhances prolactin sensitivity. In our study, we observed increased offspring viability in both reproductively experienced groups, although we still observed a lower offspring survival rate in HFD animals. Nonetheless, the differences in milk production and maternal behaviors between control and HFD groups were no longer observed. Thus, part of the prolactin resistance presented by HFD females was possibly overcome by reproductive experience leading to an improvement in lactation performance. Notably, control females had a relatively high offspring loss in the first lactation attempt. Because the protocol to induce obesity takes several months, and the control group was composed of age-matched animals, the mice started to breed at only 6 months of age. Therefore, this advanced age may have caused a decreased capacity to sustain the litters.

As mentioned before, high prolactin levels may contribute to increased food intake during pregnancy and lactation^17^. It is thought that high prolactin levels induce leptin resistance, which in turn favors the hyperphagia that occurs during these periods^17^. The reduced prolactin action on the hypothalamus of HFD females may have prevented the development of lactation-induced leptin resistance, thus leading to a lower food intake. Consequently, a shift in body weight occurred in HFD females during lactation compared to control animals. Interestingly, brain-specific STAT5 deletion, which blunts the activation of the major signaling pathway recruited by the PrlR, also causes a lower food intake during lactation^48^. Therefore, diet-induced obesity recapitulated the food intake phenotype of brain-specific STAT5 deletion, supporting the hypothesis of impaired prolactin signaling in the brain of HFD animals.

The obesity-associated factors responsible for causing high pSTAT5 basal levels in the mammary tissue and hypothalamus of HFD females are unknown. Because leptin causes STAT5 phosphorylation^49^,^50^, hyperleptinemia is a possible cause. Nonetheless, STAT5 signaling is also recruited by other growth factors and cytokines such as growth hormone, granulocyte macrophage colony-stimulating factor, interleukin-2, interleukin-3, thrombopoietin and erythropoietin^51^. Additionally, STAT5 phosphorylation is regulated by several SOCS proteins and PTPs. Increased SOCS cellular levels prevent STAT5 phosphorylation by cytokine receptors, whereas PTPs catalyze the dephosphorylation of STAT5 proteins. We assessed the expression of SOCS proteins and PTPs that interfere with PrlR signaling, and we observed reduced PTP1B mRNA levels in the mammary tissue of HFD females. Interestingly, PTP1B specifically dephosphorylates and deactivates prolactin-activated STAT5a and STAT5b proteins^52^. In addition, PTP1B is an essential regulator of alveologenesis and lactogenesis^53^. Therefore, our findings reinforce the potential importance of PTP1B in the regulation of mammary gland functions. In the hypothalamus, previous reports have shown inhibition of leptin signaling by PTP1B in obese animals^54^. As we assessed the gene expression using the whole hypothalamus, we may have missed possible changes in PTP1B levels in specific populations of prolactin-responsive neurons of lactating HFD mice.

The role of leptin in lactation performance and mammary gland functions is still unclear. Leptin treatment can rescue the fertility of ob/ob females, but these mice fail to secrete milk and feed the offspring in their first lactation^55^. Mice lacking leptin-dependent STAT3 signaling also exhibit defects in mammary ductal growth^56^. Therefore, leptin signaling during development is required for normal lactation. Other studies described the presence of LepR in mammary epithelial cells^40^,^41^,^42^. Li *et al.*^41^ showed that leptin recruits different signaling pathways to modulate the proliferation and differentiation of ductal epithelial cells, to induce the expression of milk proteins (e.g., β-casein) and to signal involution. Herein, we identified basal/myoepithelial cells as the major population of leptin-responsive cells in the mouse mammary gland. Classically, myoepithelial cell contraction is important for the milk ejection reflex. Suckling and other sensory stimuli trigger oxytocin secretion in neurons of the paraventricular nucleus of the hypothalamus (PVH) and supraoptic nucleus (SO). Then, oxytocin will be released into the blood by the posterior pituitary gland and cause myoepithelial cell contraction, leading to milk ejection. Therefore, based on our findings, leptin may regulate milk ejection through its direct actions on myoepithelial cells. Interestingly, leptin is also able to activate oxytocin neurons in the PVH and SO^57^,^58^. In addition, oxytocin mediates some of the leptin-induced attenuation of food intake^59^. Therefore, leptin may modulate central and peripheral oxytocin functions.

For leptin to regulate prolactin functions in the mammary gland, it is expected that these hormones signal to the same cell populations. Nonetheless, we demonstrated in the present study that basal/myoepithelial cells are the primary target of leptin in the mouse mammary gland, whereas prolactin acts mainly in luminal (ductal and alveolar) cells. However, Forster *et al.*^46^ recently described the existence of a paracrine basal-to-luminal endocrine axis that is critical to controlling luminal progenitor function and lactation. In this study, the authors showed that the transcription factor p63 directly regulates the expression of NRG1 by basal/myoepithelial cells. NRG1 acts paracrinally and is required for luminal ERBB4/STAT5a activation and consequent luminal progenitor cell maturation^46^. We found altered mRNA levels of ERBB4 and NRG1 in the mammary tissue of lactating HFD females. Although more studies are still necessary to investigate the specific effects of leptin on functions regulated by prolactin, our findings indicate that the effects of leptin on basal/myoepithelial cells may compromise mammopoiesis and milk production by disturbing mammary gland paracrine basal-to-luminal circuits.

In summary, our findings reveal a novel mechanism responsible for reduced lactation success in obesity, and they support a model in which high leptin levels are a possible cause of the peripheral and central prolactin resistance that is observed in obese animals. The identification of a mechanism involved in reduced lactation performance may help develop therapies that aim to overcome prolactin resistance in obese individuals. In addition, the awareness of this problem favors the promotion of initiatives to better prepare obese women for some difficulties that they may face during breastfeeding. Importantly, increasing breastfeeding success produces numerous short- and long-term health benefits for nursing mothers and their babies. These potential benefits to public health are worth the research investment in this area.

## Methods

### Animals

Female, adult wild-type (C57BL/6) and ob/ob mice were produced and maintained in standard conditions of light (12-h light/ dark cycle), temperature (22 ± 2 °C) and relative humidity (55 ± 15%). All animal procedures were approved by the Ethics Committee on the Use of Animals of the Institute of Biomedical Sciences, University of São Paulo and were performed according to the ethical guidelines adopted by the Brazilian College of Animal Experimentation.

### HFD-induced obesity

Eight-week-old female C57BL/6 mice were divided into two groups: control (normal chow; 2.99 kcal/g; 9.4% kcal derived from fat; Quimtia, Brazil; *n* = 34) and HFD (5.31 kcal/g; 58% kcal derived from fat; Pragsoluções, Brazil; *n* = 44) groups. The mice were maintained on their respective diets for at least 12 weeks before further participation in the experiment. During this period, control and HFD groups were weighed every week. Then, a GTT (2 g glucose/kg; i.p.) was performed in a subgroup of animals (*n* = 8–12/group).

### Evaluation of lactation, postpartum maternal behavior and offspring growth

Virgin control and HFD females were bred with sexually experienced C57BL/6 males. The groups were kept on their respective diets throughout all experiments. After confirming the pregnancy, mice were single-housed and monitored daily to determine the day of birth, which was considered day 1 of lactation (L1). Their body weight and food intake were determined at L1, L2, L5, L8 and L10. To guarantee comparable metabolic demands during lactation, we standardized 5 pups per litter at L2. The offspring weight was determined at L2, L5, L8 and L10. Postpartum maternal behaviors were assessed at L5 and L8. Before the behavioral test, the litter (5 pups) was weighted and separated from the mother for 4 h. Then, the litter was weighted again and distributed in the corners of the female’s cage. We evaluated the time required to contact, retrieve and group the pups into the nest and to crouch over them. After 1 h from the beginning of the test, the offspring were weighted, and the difference between the beginning and the end of the test represented the milk production, which was expressed as g/pup/h. The females that were unable to sustain their litters (the pups apparently died because of the lack of maternal care) were bred again to evaluate the lactation performance, postpartum maternal behavior and offspring growth in reproductively experienced mice. If these females were not able to sustain their litter again, then they were discarded.

### Tissue processing

Lactating control and HFD females were euthanized by decapitation at the middle of the light phase at L10. The females had free access to food and were kept with their litters until the time of euthanasia. Serum leptin levels were analyzed by ELISA (Crystal Chem.; detection limit of 0.2 ng/mL). The mammary tissue (inguinal gland) and the hypothalamus were quickly dissected and collected for posterior gene expression analyses using a protocol described previously^22^,^48^. The mammary tissue was also fixed in 4% paraformaldehyde and processed for hematoxylin-eosin staining. ImageJ software (http://rsb.info.nih.gov/ij/) was used for image analysis. The hematoxylin-eosin channels were separated by the color deconvolution plugin, as previously described^60^.

### Evaluation of prolactin responsiveness

To assess the response to an acute prolactin stimulus, virgin wild-type females (control and HFD) and ob/ob females received an intraperitoneal injection of ovine prolactin (5 μg/g; Sigma) or saline and were anesthetized 90 min later. The inguinal mammary gland was quickly collected and frozen at −80 °C. Subsequently, the females were transcardially perfused with saline followed by a 10% buffered formalin solution. Brains were collected and post-fixed in the same fixative for 1–2 h and cryoprotected overnight at 4 °C in 0.1 M PBS with 20% sucrose. Brains were cut (30-μm thick sections) in the frontal plane using a freezing microtome. Four series of tissue were collected in antifreeze solution and stored at –20 °C. The mammary tissue was processed to detect pSTAT5 levels by western blot. Brain sections were used to visualize pSTAT5 immunoreactive cells in specific hypothalamic nuclei through immunoperoxidase reaction.

### Evaluation of leptin responsiveness

To determine the leptin responsiveness, control virgin C57BL/6 females received an acute i.p. injection of mouse recombinant leptin (2.5 μg leptin/g; from Dr. A.F. Parlow, National Hormone and Peptide Program, National Institute of Diabetes and Digestive and Kidney Diseases; *n* = 5) or saline (*n* = 5), and the inguinal mammary gland and the hypothalamus (positive control) were quickly collected to detect STAT3 phosphorylation through western blot techniques.

### Histological localization of leptin receptors in mammary tissue

We used the LepR-reporter mouse to visualize LepR-expressing cells. LepR-reporter mice were generated by breeding the LepRb-IRES-Cre mouse (B6.129-Leprtm2(cre)Rck/J, Jackson Laboratories) with the Cre-inducible tdTomato-reporter mouse (B6;129S6-Gt(ROSA)26Sortm9(CAG-tdTomato)Hze/J, Jackson Laboratories). This mouse model has been validated^6^,^45^. Only cells that express the long form of LepR contain the red fluorescent tdTomato protein in the LepR-reporter mouse. To identify the localization of LepR-expressing cells in the mammary tissue, the inguinal glands of four pregnant LepR-reporter mice were dissected at specific developmental time-points (16–18 days of pregnancy). The tissue was fixed in 4% paraformaldehyde overnight at 4 °C and processed for hematoxylin-eosin staining or immunostaining according to the following procedures: 4-μm sections were deparaffinized, rehydrated, and subjected to 10 mM citrate buffer pH 6,0 antigen retrieval for 30 min at 95 °C. Anti-tdTomato (Clontech) or Anti-CK5 (Abcam) primary antibodies were incubated overnight at 4 °C at 1:200 dilution, and Alexa fluor 488-conjugated and Alexa fluor 633-conjugated secondary antibodies were used, respectively, at 1:750 dilutions. Samples were mounted in Prolong Gold antifade reagent with DAPI (Molecular Probes).

### Western blotting

Immediately after collection, the hypothalamus and mammary glands were homogenized in RIPA buffer (Sigma) containing a cocktail of protease and phosphatase inhibitors (1:100, Sigma) and centrifuged (14000 RPM, 4 °C for 20 minutes), and the supernatants were retained. After determining the total protein concentration (Pierce BCA Protein Assay, Thermo Scientific), 50 μg of total protein was loaded on a 10% SDS-PAGE gel, and the separated proteins were then transferred to a nitrocellulose membrane (Bio-Rad). Membranes were blocked with 5% bovine serum albumin and incubated overnight at 4 °C using commercially available primary antibodies (1:1000) to identify pSTAT3^Tyr705^ (Cell Signaling), pSTAT5^Tyr694^ (Cell Signaling), GAPDH (Santa Cruz) or α-tubulin (Cell Signaling). Next, we incubated the membranes for 45 min in 1:10,000 secondary antibody (IRDye 800CW, Li-COR). Proteins were detected by fluorescence and analyzed using the Li-COR Odyssey system (Li-COR), and protein amounts were normalized to GAPDH or α-tubulin amounts.

### Immunoperoxidase

Brain sections were rinsed in 0.02 M potassium PBS, pH 7.4 (KPBS), followed by a pretreatment in an alkaline (pH > 13) water solution containing 1% hydrogen peroxide and 1% sodium hydroxide for 20 min. After rinsing in KPBS, sections were incubated in 0.3% glycine and 0.03% lauryl sulfate for 10 min each. Next, sections were blocked in 3% normal donkey serum for 1 h, followed by incubation in anti-pSTAT5^Tyr694^ primary antibody (1:1000; Cell Signaling) for 40 h. Subsequently, sections were incubated for 1 h in biotin-conjugated secondary antibody (1:1,000, Jackson Laboratories) and next for 1 h with an avidin-biotin complex (1:500, Vector Labs). The peroxidase reaction was performed using 0.05% 3,3′-diaminobenzidine, 0.25% nickel sulfate and 0.03% hydrogen peroxide. We counted the number of pSTAT5 immunoreactive cells on one side of a representative rostrocaudal level of each area analyzed. Photomicrographs of brain sections were acquired with a Zeiss Axiocam HRc camera adapted to a Zeiss Axioimager A1 microscope (Zeiss, Munich, Germany). Images were digitized using Axiovision software (Zeiss). Photoshop image-editing software was used to combine photomicrographs into plates. Only sharpness, contrast and brightness were adjusted.

### Statistical analysis

The results are expressed as the mean ± SEM. The differences between groups were compared using an unpaired two-tailed Student’s t-test. Fisher’s exact test was used to compare the percentage of successful litters between groups. Statistical analyses were performed using GraphPad Prism software. We considered *p* values less than 0.05 to be statistically significant.

## Additional Information

**How to cite this article**: Buonfiglio, D. C. *et al.* Obesity impairs lactation performance in mice by inducing prolactin resistance. *Sci. Rep.* **6**, 22421; doi: 10.1038/srep22421 (2016).
